# Supplementary figures and images for: Autocorrelation-based method to identify disordered rhythm in Parkinson’s disease tasks: A novel approach applicable to multimodal devices
Source: PLoS One. 2020 Oct 8;15(10):e0238486. doi: 10.1371/journal.pone.0238486 (PMC7544077; doi:10.1371/journal.pone.0238486)

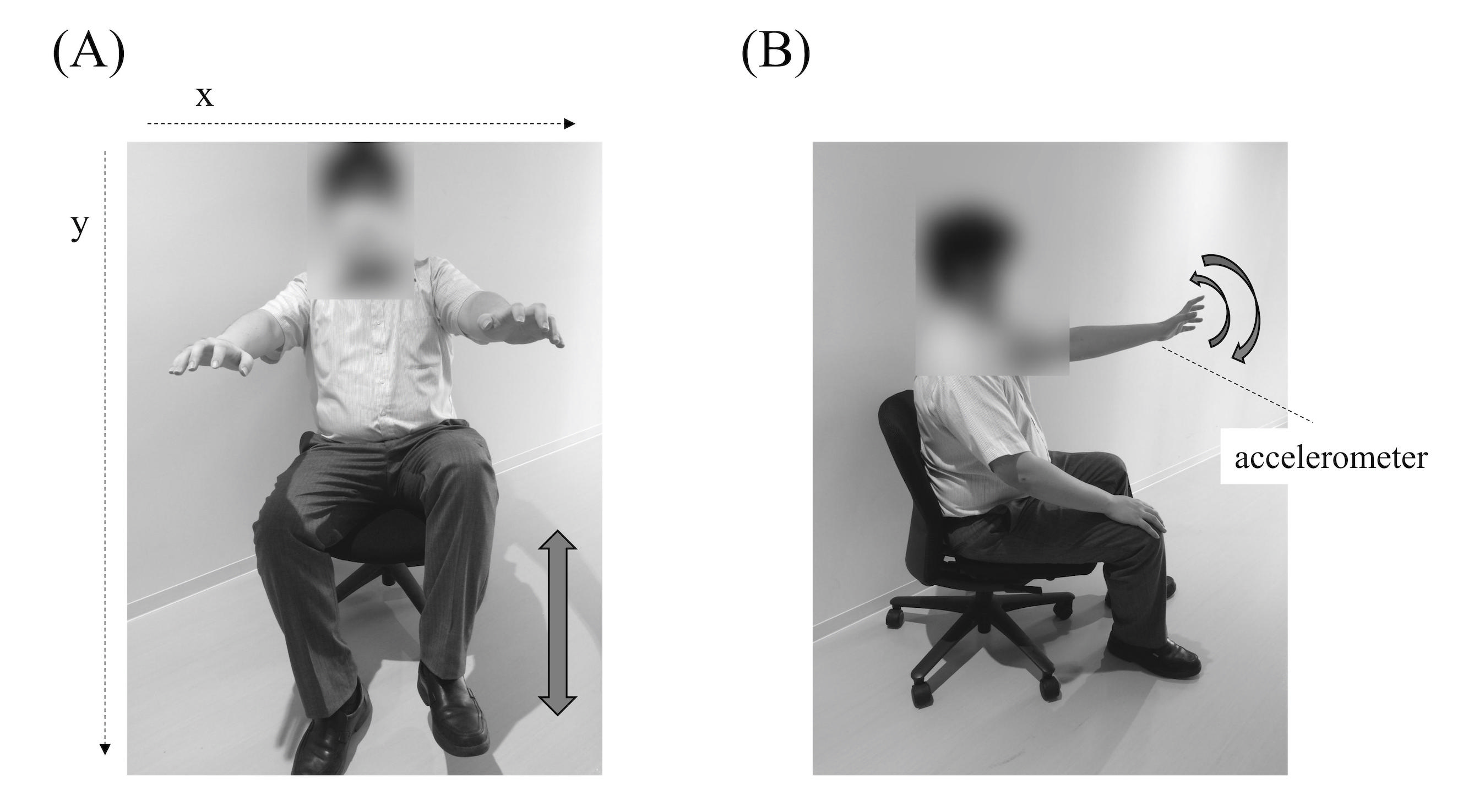

Supplement: S1 Fig — Images showing the tasks and their positions (performed by one of our authors (K.S) only for demonstration). In dataset (1) of leg agility task, the participant raises and stomps either the left or right foot while sitting on a chair as in S1A Fig (for details, see original article of the data [15]). The data is obtained as the series of 2D coordinates of knee of the tapping leg within each frame (axes outline as x and y). In dataset (2) of pronation-supination task, the participant lifts one arm into a horizontal position and turns the palm up and down 10 times while sitting on a chair (S1B Fig) (for details, see original article of the data [7]). The data is obtained as the serial 3-axis acceleration of the accelerometer attached to the wrist of the ipsilateral arm of the task. (TIF) [file pone.0238486.s001.tif]
